# Assessment of Oral Health-Related Quality of Life of the United Arab Emirates’ Elderly Population: Observational Prospective Cross-Sectional Study

**DOI:** 10.3390/dj13030123

**Published:** 2025-03-11

**Authors:** Faris El-Dahiyat, Ammar Abdulrahman Jairoun, Obaida Jairoun, Islam Eljilany, Mohammed Alsbou

**Affiliations:** 1 Clinical Pharmacy Program, College of Pharmacy, Al Ain University, Al Ain P.O. Box 64141, United Arab Emirates; 2 AAU Health and Biomedical Research Center, College of Pharmacy, Al Ain University, Al Ain P.O. Box 64141, United Arab Emirates; 3 Health and Safety Department, Dubai Municipality, Dubai P.O. Box 67, United Arab Emirates; 4 College of Dentistry, Ajman University, Ajman P.O. Box 346, United Arab Emirates; o.jairoun@ajman.com; 5 Departments of Cutaneous Oncology, H. Lee Moffitt Cancer Center and Research Institute, Tampa, FL 33612, USA; 6 Faculty of Medicine, Ajman University, Ajman P.O. Box 346, United Arab Emirates

**Keywords:** GOHAI, oral health, quality of life, elderly, HRQoL

## Abstract

**Background/Objectives:** The current study aimed to evaluate the oral health self-perception on quality of life in the elderly using the Geriatric Oral Health Assessment Index (GOHAI) to assess the impact of demographic and oral health factors on oral health-related quality of life. **Methods:** An observational prospective cross-sectional study for the entire six-month period was conducted in a dental health care center in the United Arab Emirates. The principal inclusion criterion is being aged 60 and over. The GOHAI questionnaire is composed of 4 domains of 12 items that address functional limitation, pain and discomfort, psychological impacts, and behavioral impacts. Patients were questioned about the frequency at which they experience any of the 12 listed problems. **Results:** A total of 318 respondents participated in the study and completed the whole questionnaire. Among these participants, 63.5% (*n* = 202) were male and 86.8% (*n* = 276) were ≤70 years. The average GOHAI score was 13.25, with a 95% confidence interval (CI) [12.4%, 14%], indicating a low self-perception of oral health by the allocated sample. Statistical modeling identified dry mouth (OR = 2.21, 95% CI 1.40–3.48) and chewing problems (OR = 1.87, 95% CI 1.09–3.20) as the strongest determinants of poor oral health-related quality of life (OHRQoL) in the elderly population. **Conclusions:** Healthcare professionals should develop targeted strategies to address the specific needs of this population, ensuring sustained improvements in their quality of life.

## 1. Introduction

Older people’s health-related quality of life (HRQoL) has received much attention from healthcare professionals (HCPs) in recent years since many older people are unable to practice an essential oral health and hygiene routine [1,2,3,4]. Generally, oral HRQoL (OHRQoL) is considered a fundamental element within HRQoL. It is defined as the degree to which oral conditions prevent people from functioning normally [5,6,7]. Also, the World Health Organization (WHO) has recognized the importance of OHRQoL by incorporating it into its Global Oral Health Program [8,9,10]. The concept is multifaceted, as oral health is connected to the quality of life (QoL) in multiple ways. Oral health impacts mental, psychological, social, and physical well-being and development, including communicative and alimentary impairment [3,9,11,12,13]. This has been shown across a wide range of practical and theoretical work.

Many studies that aim to assess disorders often prioritize clinical factors over patients’ self-perceptions. To rectify this imbalance, several Oral Health-Related Quality of Life (OHRQoL) questionnaires have been developed. Notable examples include the Oral Health Impact Profile (OHIP), Oral Impacts on Daily Performance (OIDP), and Geriatric Oral Health Assessment Index (GOHAI) [14,15,16,17].

The GOHAI, pioneered by Atchison and Dolan [18], was the first tool designed to bridge the gap between clinical data and older adults’ personal perspectives on their dental-related physical, physiological, and psychological concerns. Its purpose is to enhance our understanding of how oral health impacts individuals by incorporating patients’ subjective experiences alongside clinical assessments. This approach aims to provide a more comprehensive insight into the effects of oral health on people’s lives.

Globally, as life expectancy increases, there is a visible demographic change towards an older (60+) population. The United Arab Emirates (UAE) is no exception; therefore, HCPs should plan around the needs of this population in order to ensure their ongoing QoL and their HRQoL. The current article uses the GOHAI, which evaluates patients’ self-perceptions of their oral health, to investigate public health needs related explicitly to the OHRQoL of an aging population. This study aimed to evaluate the oral health self-perception on quality of life in the elderly using the GOHAI index in order to assess the impact of demographic and oral health factors on OHRQoL.

## 2. Methods

### 2.1. Study Setting, Design, and Population

This prospective observational cross-sectional study was conducted in the Sharjah district of the United Arab Emirates (UAE) at a dentistry clinic. The study, which was conducted from January to June 2019, exclusively focused on patients aged 60 years and older who sought consultations at this clinic. The exclusion criteria were expanded to include individuals under 60 years of age, those with cognitive impairments or severe communication difficulties, adults with acute medical conditions requiring immediate care, and patients who declined to provide informed consent.

The initial page of the survey contained an invitation detailing the study’s objectives, followed by the informed consent form on the second page. Study participants provided their consent through signature on the consent form, facilitating data collection via convenient sampling during in-person interviews conducted by one of the study investigators.

The dentistry healthcare clinic was strategically located in the city center, offering convenient access to public transportation. This dental healthcare clinic is located in the heart of Sharjah City, UAE. Primarily serving outpatients, the facility offers advanced dental procedures through four modern dental units, staffed by experienced practitioners. The clinic provides accessible and comprehensive oral health solutions tailored to meet the diverse needs of its clientele, including older patients. While the majority of attendees come from middle-income backgrounds, the clinic also serves individuals from various socioeconomic and educational contexts, resulting in a reasonably heterogeneous sample representative of both urban and suburban populations. The research adhered rigorously to ethical principles in accordance with the guidelines specified in the World Medical Association Declaration of Helsinki (2008 version). Additionally, the study received approval from the ethics and health committee of the health center.

### 2.2. Rationale for Study Design

This research utilized a prospective observational cross-sectional design to analyze oral health-related quality of life (OHRQoL) among older people at a specific point in time. The design is particularly suited for identifying connections between demographic features, oral health aspects, and self-perceived OHRQoL, as it enables the efficient collection of extensive data sets without requiring ongoing oversight, making it an effective approach for generating hypotheses and establishing foundational data for longitudinal studies in similar populations.

### 2.3. Participant Recruitment

Participants were recruited through a purposive sampling approach at a Sharjah, UAE dental healthcare center between January and June 2019. Eligibility was limited to individuals over the age of 60, who possessed the mental capacity to provide informed consent and could travel to the center within the specified timeframe. On the day of attendance, potential participants were approached by an experienced research investigator, who explained the study’s purpose and procedures. Those willing to participate were provided with an invitation and consent documents. Written informed consent was obtained from all participants prior to data collection, ensuring their participation was voluntary. Confidentiality was maintained by removing all identifying information from the data records.

### 2.4. Sample Size Calculation

The sample size was computed using the Roasoft online calculator (www.roasoft.com), presuming the average population above 60 years old who visit the clinic is 2000. A sample of 318 participants was seen to be enough to obtain significant results, achieve a 95% confidence interval with a 5% marginal error, and 50% response distribution.

### 2.5. Survey Development

The GOHAI questionnaire was used as a tool in this survey (appendix). Information regarding gender, age, marital and socioeconomic status, lifestyle, and education was collected. The GOHAI questionnaire is composed of 4 domains of 12 items that address functional limitation, pain and discomfort, psychological impacts, and behavioral impacts. Patients were asked about the frequency at which they encounter any of 12 listed problems, using a 5-point Likert scale rating (0 = ‘never’ or 1 = ‘sometimes’, 2 = ‘Fairly often’, 3 = ‘very often’, 4 = ‘all the time’). Afterward, a dental surgeon performed oral examinations, and oral health statuses were collected under the World Health Organization protocol [19].

### 2.6. GOHAI Questionnaire Validation Process

To ensure validity and reliability in the application of the Geriatric Oral Health Assessment Index (GOHAI), a thorough validation process was conducted. This process was overseen by two dental experts from Ajman University, who reviewed the questionnaire for clarity, relevance, and comprehensiveness. Their feedback prompted minor adjustments to enhance the tool’s contextual appropriateness and cultural sensitivity. While the original validation did not include input from older patients, future research will address this limitation to better align the questionnaire with the lived experiences of the target population.

Initially available only in English, the GOHAI questionnaire was adapted into an Arabic version to reflect the cultural and linguistic needs of older adults in the UAE. A standardized forward-backward translation method was employed to preserve the material’s equivalence and accuracy. The translated version was reviewed and approved by the dental experts to ensure clarity and suitability for the target demographic. While a fully validated Arabic version of GOHAI exists—developed by Daradkeh and Khader (2008) [20]—it was validated exclusively in North Jordan. Due to the significant linguistic and cultural differences across Arabic-speaking areas, we concluded that there was a need to adapt the tool to accurately reflect the dialects, expressions, and cultural context of the UAE population.

Oral health-related quality of life (OHRQoL) is influenced by local healthcare access and socio-cultural factors. As such, population-specific adaptations are required. Our partial validation process was specifically focused on key psychometric properties, including internal consistency and face validity, confirming the reliability of the adapted instrument within our study context.

### 2.7. Oral Health Investigation Procedure

After completing the questionnaires, each participant underwent an oral health examination conducted by a licensed dental surgeon. The examinations adhered to the World Health Organization (WHO) guidelines for oral health assessments to ensure data reliability and comparability. The procedure included a systematic evaluation of dental status (dentate or edentulous), recording any missing teeth, prosthetic rehabilitation needs, and additional factors such as dry mouth or chewing difficulties. All data collected during the examinations were documented on standardized forms and subsequently cross-checked by a second investigator to ensure accuracy.

### 2.8. Study Variables

GOHAI scores serve as the dependent variable in this study, while the independent variables encompass a range of oral health characteristics (such as dental status, number of missing teeth, need for prosthetics, chewing difficulties, dry mouth, and self-rated oral health) and demographic factors (including gender, age, marital status, education level, and lifestyle).

To comprehensively address potential unmeasured confounding variables, our research employed a multifaceted approach. This approach encompassed factors like comorbidities (chronic illnesses), cultural influences (beliefs and practices related to oral health), dietary habits (impact of diet on oral health and nutrient intake), and medication usage (types and frequency). During the study’s design phase, we identified significant confounders through an extensive literature review and expert consultation to guide our data collection process, ensuring the acquisition of relevant information.

Additionally, we conducted subgroup analyses to investigate variations in the effects of major factors on oral health-related quality of life across different demographic and health parameters. In our commitment to transparent reporting, we recognize the importance of acknowledging any limitations associated with unmeasured confounders to ensure a careful interpretation of the study’s results.

### 2.9. Statistical Analysis

The data were collected in an Excel sheet, then transferred and analyzed using the SPSS software version 23 (IBM, New York, NY, USA). The GOHAI scores were calculated by adding response codes for all 12 items. Scores could thus range from a maximum of 48 to a minimum of 0, with higher scores indicating poorer OHRQoL. In this study, 4 cut-off limits were used for grading the oral health quality of life based on the 25th, 50th, and 75th quartiles [21,22,23,24]. The perception of oral health quality is categorized as good (score: 0–9), moderate (score: 10–12), low (score: 13–17), and poor (score: ≥18). The descriptive analysis was performed. Categorical data and socio-economic characteristics were presented as frequency and percentage, while continuous data were presented as mean and standard deviation (SD). A simple and multiple binary logistic regression was performed to examine the association between the GOHAI score and other significant risk factors. The stepwise method was applied for variable selection and model building. The odds ratios show the magnitude of the association, and their corresponding *p*-values indicate whether the association is statistically significant or not. A two-tailed *p*-value < 0.05 was chosen as the criteria to make decisions regarding statistical significance.

### 2.10. Ethical Consideration

The research adhered to the ethical standards outlined in the Declaration of Helsinki (2008 version). Ethical review and research approval were obtained from Al Luluah Al Baidhaa Dental Clinic and the Al Ain University Research Ethics Committee (Approval Code: COP/2019/22, Approval Date: 5 January 2019). Participants were provided with clear information about their rights, including the ability to withdraw from the study at any time without consequences, as well as assurances of anonymity and confidentiality. Written informed consent was obtained from all contributors prior to their participation.

To ensure data security, encrypted electronic data were stored within password-protected systems accessible only to authorized personnel. Physical documentation, such as completed surveys and consent forms, was securely stored in locked cabinets. All data will be retained for a period of five years following publication of the research, after which it will be securely destroyed.

## 3. Results

### 3.1. Demographic and Socio-Economic Characteristics

Over six months, a total of 500 participants were approached, among whom 318 respondents participated in the study and completed the whole questionnaire (63.6% response rate).

The majority of participants, 63.5% (*n* = 202), were male, and plenty, 86.8% (*n* = 276), were ≤70 years. The marital status revealed 89 single, 96 divorced, 78 widowed, and 55 married. Most of the participants (88.7%) have less than a school education. According to the lifestyle, around one half (48.7%) live in a familiar environment, and the other half (51.3%) live alone. (Table 1).

### 3.2. Oral Health Characteristics

From an oral health perspective, many participants (88.1%) were dentate, and 35.2% of them had more than 20 missing teeth. Regarding oral rehabilitation, 29.9% of the subjects needed a removable prosthesis, whereas 70.1% did not need prosthesis rehabilitation. A quarter (23.6%) of the total participants had chewing problems, while dry mouth was reported by 141 (44.3%). When they were asked to rate their oral health, good oral health accounted for 48.7% of the sample; however, 51.3% indicated terrible oral health. Table 1 shows the oral health characteristics of the participants.

### 3.3. Assessment of Self-Perceived Oral Health Status

The mean ± SD of the GOHAI score was 13.25 ± 7.3 with a 95% confidence interval (CI) [12.4%, 14%]. Using the grading mentioned above, 9.9% (*n* = 95) perceived their oral health as good, 24.2% (*n* = 77) as moderate, 22.3% (*n* = 71) as low, and 23.6% (*n* = 75) as poor. (Table 2).

Using GOHAI ADD scores, 12.6% reported no functional limitations, 7.9% no pain or discomfort, 6.6% no psychological impacts, and 15.7% no behavioral impacts. Overall, only 11 participants (3.5%) did not show any issues on the GOHAI scale. Table 3 presents the responses for GOHAI items.

### 3.4. Factors Associated with Self-Perceived Oral Health Status

Table 4 displays the results of the GOHAI that are equal to or above the median score (GOHAI ≥ 12). Dental status, oral rehabilitation, chewing problems, and self-rated oral health had statistically significant relations with GOHAI (*p* = 0.01, *p* = 0.004, *p* = 0.023, *p* = 0.001, *p* = 0.006, respectively).

Edentulous participants had a 2.68 times greater risk of having GOHAI ≥ 12 compared to dentate participants (95% CI 1.26–5.73). Similarly, subjects who required prosthesis rehabilitation had a 2.09 times greater risk of experiencing GOHAI ≥ 12 compared to those who did not require it (95% CI 1.27–3.46).

Moreover, participants with chewing problems had a 1.87 times greater risk of reporting a poorer perception of oral health (GOHAI ≥ 12) compared to those without chewing problems (95% CI 1.09–3.20). Subjects with dry mouth had a 2.21 times greater risk of reporting a poorer perception of oral health (GOHAI ≥ 12) compared to those without dry mouth (95% CI 1.40–3.48). Furthermore, participants with bad self-rated oral health had a 1.88 times greater risk of having GOHAI ≥ 12 compared to those with self-rated oral health (95% CI 1.20–2.93). Stepwise logistic regression analysis proved that dry mouth and chewing problems are the only variables that remained associated significantly with GOHAI. For more details, see Table 4.

## 4. Discussion

The primary aim of this study was to assess the OHRQoL among the geriatric population in the UAE. A total of 318 participants were recruited and evaluated for their perceived oral health and oral health-related quality of life. About half of the sample reported bad self-rated oral health. Just under one-third (29.9%) of the sample perceived their oral health as good, whereas about half of the sample reported either poor or low perception of their oral health on the GOHAI scale. The significant factors that affected the oral-related quality of life in this sample were dental status, oral rehabilitation, chewing problems, dry mouth, and self-rated oral health. The mean ± SD of the GOHAI score was 13.25 ± 7.3, which means that on a scale of 0 to 100, participants scored on average 27.6 points in GOHAI, indicating a low self-perception of oral health by the allocated sample.

Oral health-related quality of life is consistently perceived as an essential part of the general quality of health and well-being [18,25]. This importance is more pronounced in the elderly, who are more likely to have comorbid diseases and quality of life-related issues [4,23,26,27,28]. In the present study, most of the patients experienced suboptimal OHRQoL. These findings are consistent with those from Iinuma et al. [29], who used the same OHRQoL in an elderly Japanese sample. Similarly, using OHIP-14, which is a different OHRQoL assessment tool, the elderly from China [27], Spain [26], and the USA [30] have been found to have low levels of OHRQoL.

Our data have identified dental status as a significant determinant of OHRQoL. This is consistent with the findings reported by Sheng et al. [7]. These results are in agreement with Kohli et al. [30], who identified an association between the dental status described as decay, missing, and filled teeth (DMFT) and OHRQoL measured using OHIP-14. These findings are further supported by a study from Spain, which showed a relation between OHRQoL and dental status. Collectively, these findings highlight the need for improving dental status to increase OHRQoL. This requires community-based interventions that target the elderly population and increase their awareness about the importance of oral health.

OHRQoL has been shown to affect the overall quality of life. For instance, low OHRQoL will negatively affect the overall quality of life. The magnitude of this problem will be continuously increasing with the aging of the population. It will result in a sizeable increase in health care costs and lower levels of quality of life [18]. These consequences will present a severe challenge for healthcare decision-makers in the upcoming days.

To the best of our knowledge, this study is the first assessment of the OHRQoL in the UAE and the Middle East. Although the UAE has a predominantly young population, older adults represent a distinct demographic with unique health needs and challenges. Furthermore, like other regions of the world, the percentage of elderly in this region is increasing. This represents a challenge and an opportunity for health decision-making. Early intervention aiming at assessing and improving quality of life will reduce the detrimental effect of health issues in the elderly and will minimize the need for more costly interventions that will be needed at later stages of life. This research contributes to the existing body of knowledge by highlighting the unique challenges faced by older adults in the UAE regarding oral health. Our findings are in agreement with studies from Western and Asian regions; however, this research contributes to the literature by addressing oral health challenges in a relatively under-researched Middle Eastern geriatric population, which has distinct healthcare access dynamics and cultural perceptions. Additionally, identifying modifiable factors such as dry mouth and chewing difficulties provides actionable opportunities for interventions, paving the way for improved OHRQoL among a growing demographic.

When discussing the limitations of our research, it is important to acknowledge a regrettable oversight in our questionnaire validation procedure: the omission of input from older patients who visit dental clinics. We recognize that firsthand experiences are pivotal in assessing questionnaire validity, and the absence of this particular demographic limits the comprehensiveness of our results. To enhance the instrument’s relevance and applicability, it is imperative to ensure a comprehensive validation process that encompasses diverse perspectives.

Nevertheless, our research remains focused on assessing the self-perceived oral health of the elderly and its correlation with their quality of life. To this end, we utilize the Geriatric Oral Health Assessment Index (GOHAI) to investigate how both demographic and oral health variables impact oral health-related quality of life. We highly value the input of our reviewers and pledge to incorporate their insightful recommendations into our future research endeavors.

This research has several limitations that should be considered when interpreting the results. Firstly, recruiting participants from a dental facility may introduce selection bias, as individuals with existing oral health concerns are likely to be overrepresented. This factor may limit the generalizability of the findings to the broader older population. Future studies may benefit from employing community-based recruitment methods to capture a wider range of oral health experiences, including those of individuals who do not regularly seek dental care.

Secondly, while the GOHAI questionnaire was transculturally adapted into Arabic and validated, the lack of input from older patients during the validation process may have influenced the results.

Thirdly, the cross-sectional design only captures data at a single point in time, preventing the establishment of causal relationships between oral health and OHRQoL.

Lastly, unmeasured confounding factors—such as medication use and dietary habits—may have affected the findings, despite efforts to control for key known variables.

## 5. Conclusions

This study shows that factors including dry mouth, chewing issues, and self-rated oral health have a significant impact on the oral health-related quality of life (OHRQoL) of the older population in the United Arab Emirates. The need for focused oral health interventions is highlighted by the mean GOHAI score of 13.25, which indicates a poor self-perception of oral health. The current study highlights how crucial it is to address the psychological effects and functional limits related to oral health in order to enhance general well-being in elderly populations. Health authorities’ interventions, such as conducting awareness campaigns, will contribute to increasing public awareness of oral health-related quality of life.

## Figures and Tables

**Table 1:** Frequency table for demographic and oral health characteristics (*n* = 318).

Demographic and Socio-Economic Characteristics			
Demographic Characteristics	Response	Frequency	Percentage (%)
Gender	Male	202	63.5%
	Female	116	36.5%
Age	≤70 years	276	86.8%
	>70 years	42	13.2%
Marital Status	Single	89	28%
	Divorced	96	30.2%
	Widower	78	24.5%
	Married	55	17.3%
Education level	Less than school	282	88.7%
	High school and college	36	11.3%
Lifestyle	Family environment	155	48.7%
	Living alone	163	51.3%
Oral health characteristics			
Dental status	Dentate	280	88.1%
	Edentulous	38	11.9%
Number of missing teeth	≤20	206	64.8%
	>20	112	35.2%
Prosthesis required	Yes	95	29.9%
	No	223	70.1%
Chewing problems	Yes	75	23.6%
	No	243	76.4%
Dry mouth	Yes	141	44.3%
	No	177	55.7%
Self-rated oral health	Good	155	48.7%
	Bad	163	51.3%

**Table 2:** Descriptive statistics for the GOHAI score.

Statistics	GOHAI ADD Score
Range	0–37
% with a score of Zero	3.5%
Mean (SD)	13.25 (7.3)
Median	12
Percentiles	
25	9
50	12
75	17
GOHAI grading	N (%)
Good perception (0–9)	95 (29.9%)
Moderate perception (10–12)	77 (24.2%)
Low perception (13–17)	71 (22.3%)
Bad perception (≥18)	75 (23.6%)
GOHAI, Geriatric Oral Health Assessment, SD, Standard deviation Score of zero: subject did not show any issues.	

**Table 3:** Frequency table for the responses for GOHAI items (*n* = 318).

GOHAI Items	Never	Sometimes	Fairly Often	Very Often	All the Time (%)
Functional limitation					
Trouble biting/chewing food	92 (28.9%)	157 (49.4%)	37 (11.6%)	23 (7.2%)	9 (2.8%)
Uncomfortable to swallow	103 (32.4%)	154 (48.4%)	33 (10.4%)	22 (6.9%)	6 (1.9%)
Prevented from speaking	67 (21.1%)	155 (48.7%)	60 (18.9%)	28 (8.8%)	8 (2.5%)
Pain and discomfort					
Discomfort when eating	47 (14.8%)	166 (52.2%)	52 (16.4%)	41 (12.9%)	12 (3.8%)
Use medication to relieve pain	117 (36.8%)	138 (43.4%)	44 (13.8%)	18 (5.7%)	1 (0.3%)
Teeth, gums, sensitive to hot/cold	96 (30.2%)	142 (44.7%)	52 (16.4%)	23 (7.2%)	5 (1.6%)
Psychological impacts					
Unhappy with appearance	84 (26.4%)	173 (54.4%)	47 (14.8%)	11 (3.5%)	3 (0.9%)
Worried or concerned	74 (23.3%)	147 (46.2%)	53 (16.7%)	28 (8.8%)	16 (5%)
Nervous or self-conscious	66 (20.8%)	143 (45%)	63 (19.8%)	34 (10.7%)	12 (3.8%)
Behavioral impacts					
Uncomfortable eating in front of people	131 (41.2%)	116 (36.5%)	39 (12.3%)	24 (7.5%)	8 (2.5%)
Limit kinds or amounts of food	87 (27.4%)	158 (49.7%)	38 (11.9%)	24 (7.5%)	11 (3.5%)
Limit contact with others	87 (27.4%)	157 (49.4%)	47 (14.8%)	21 (6.6%)	6 (1.9%)
GOHAI, Geriatric Oral Health Assessment; SD, Standard deviation					

**Table 4:** Regression model applied to each demographic and oral health variable and factors associated with GOHAI.

Demographic	GOHAI ≥ 12			
	OR	95% CI		*p*-Value *
Major (Ref. male)				
Female	1.09	0.69	1.73	0.705
Age (Ref. ≤ 70 years)				
≤70 years	1.170	0.61	2.25	0.639
Marital status (Ref. Married)				
Single	1.69	0.86	3.32	0.131
Divorced	1.68	0.86	3.28	0.128
Widower	1.14	0.57	2.28	0.711
Lifestyle (Ref. Family environment)				
Living alone	1.38	0.89	2.15	0.154
Dental status (Ref. Dentate)				
Edentulous	2.68	1.26	5.73	0.01
Number of missing teeth (Ref. ≤ 20)				
>20	1.31	0.820	2.07	0.26
Prosthesis required (Ref. No)				
Yes	2.09	1.27	3.46	0.004
Chewing problems (Ref. No)				
Yes	1.87	1.09	3.20	0.023
Dry mouth (Ref. No)				
Yes	2.21	1.40	3.48	0.001
Self-rated oral health (Ref. Good)				
Bad	1.88	1.20	2.93	0.006
Independent variables factors associated with GOHAI				
Dry mouth	2.164	1.358	3.447	0.001
Chewing problems	1.757	1.007	3.066	0.047

OR, odds ratio; CI, confidence interval; GOHAI ≥ 12, median split. * *p*-value < 0.5.

## Data Availability

The data presented in this study are available on request from the corresponding author due to ethical and privacy considerations, access to the data is restricted and will be granted upon reasonable request for research purposes.
